# The role of nanotechnology-based approaches for clinical infectious diseases and public health

**DOI:** 10.3389/fbioe.2023.1146252

**Published:** 2023-04-03

**Authors:** Xuefang Chen, Jinfang Xu, Bangju Ji, Xingliang Fang, Ketao Jin, Jun Qian

**Affiliations:** ^1^ Department of Clinical Laboratory, Affiliated Hospital of Shaoxing University, Shaoxing, China; ^2^ Department of Emergency Medicine, Affiliated Hospital of Shaoxing University, Shaoxing, China; ^3^ Department of Colorectal Surgery, Shaoxing People’s Hospital, Shaoxing, China; ^4^ Department of Hepatobiliary Surgery, Affiliated Hospital of Shaoxing University, Shaoxing, China; ^5^ Department of Colorectal Surgery, Affiliated Jinhua Hospital, Zhejiang University School of Medicine, Jinhua, China; ^6^ Department of Colorectal Surgery, Xinchang People’s Hospital, Affiliated Xinchang Hospital, Wenzhou Medical University, Xinchang, China

**Keywords:** nanotechnology, antiseptic, intensive care unit, ICU, SARS-CoV-2 (2019-nCoV), bio-kil

## Abstract

Given the high incidence of infection and the growing resistance of bacterial and viral infections to the traditional antiseptic, the need for novel antiseptics is critical. Therefore, novel approaches are urgently required to reduce the activity of bacterial and viral infections. Nanotechnology is increasingly being exploited for medical purposes and is of significant interest in eliminating or limiting the activity of various pathogens. Due to the increased surface-to-volume ratio of a given mass of particles, the antimicrobial properties of some naturally occurring antibacterial materials, such as zinc and silver, increase as particle size decreases into the nanometer regime. However, the physical structure of a nanoparticle and the way it interacts with and penetrates the bacteria also appear to provide unique bactericidal mechanisms. To measure the efficacy of nanoparticles (diameter 100 nm) as antimicrobial agents, it is necessary to comprehend the range of approaches for evaluating the viability of bacteria; each of them has its advantages and disadvantages. The nanotechnology-based disinfectants and sensors for SARS-CoV-2 provide a roadmap for creating more effective sensors and disinfectants for detecting and preventing coronaviruses and other infections. Moreover, there is an increasing role of nanotechnology-based approaches in various infections, including wound healing and related infection, nosocomial infections, and various bacterial infections. To meet the demand for patient care, nanotechnology-based disinfectants need to be further advanced with optimum approaches. Herein, we review the current burden of infectious diseases with a focus on SARS-CoV-2 and bacterial infection that significantly burdens developed healthcare systems and small healthcare communities. We then highlight how nanotechnology could aid in improving existing treatment modalities and diagnosis of those infectious agents. Finally, we conclude the current development and future perspective of nanotechnology for combating infectious diseases. The overall goal is to update healthcare providers on the existing role and future of nanotechnology in tackling those common infectious diseases.

## 1 Introduction

Nanotechnology significantly benefits cellular and gene therapies for treating cancer and other life-threatening diseases (Herranz et al., 2011; Ma, 2022). Using designed nano-devices and nanostructures, full monitoring, regulating, constructing, restoration, defense, and enhancement of all biological systems in humans, beginning at the molecular level, are currently accessible in cancer (Roma-Rodrigues et al., 2020). Given the present rate of invention, it is challenging to predict what will or will not be accessible in more than 30 years. Developing nanotechnologies are maturing rapidly; we anticipate that intensive care units (ICU) will embrace them within 20–30 years (Wong, 2017). The role of nanotechnology has been rapidly increasing in the current decades (Matharu et al., 2018). The introduction of nanoscience and nanotechnology is transforming conventional medical instruments linked to a patient giving insights into intelligent systems for continuous evaluation and speedy critical care decision-making. To upgrade the prognosis of patients, it is necessary to measure the biomarkers of illness (nucleic acids, antibodies, proteins, and cells) present in deviating numbers in tissue or body fluids when illness exists (Wishart et al., 2021). Lab-on-a-chip (LOC) is one example of a compact “point-of-care (POC)” instrument that is crucial. The identification of pathogens is necessary for early diagnosis of an infection. However, the key concentration in the ailment start and development cycle is often extremely low, such as in the case of sepsis, 5–10 colony forming units (CFU) per milliliter (Schuster, 1983; Nasa et al., 2012; Martin-Loeches and Perner, 2016; Martin and Badeaux, 2017). Even though advancements have been made in POC equipment, the crucial requirements for precision and sensitivity require a centralized laboratory that adheres to strict norms and too long of a turnaround time for medical usages, such as blood culture combined with lysis and amplification of the DNA component to detect infections in sepsis cases (Zilahi et al., 2016). This poor turnaround time necessitates the ICU physician to respond without access to complete analytical data, frequently based entirely on medical observations. Speedy diagnosis and cure substantially influence patient outcomes (Perner et al., 2017). Though infections are not always straightforward to identify, particularly in seriously sick individuals, this practice has caused hospitals to overuse the medicines, contributing to high antimicrobial levels of resistance (Martin-Loeches et al., 2013). Effective identification of pathogens is necessary to prevent and cure life-threatening infections in seriously ill patients. Sepsis continues to be a leading global cause of sickness and mortality despite recent advancements in biosensor technology. Sepsis is more often the cause of death in developing countries than in any other condition. LOC instruments provide several benefits for detecting pathogens, including miniaturization, smaller sample size, mobility, quick recognition time, and POC diagnosis (Nwankire et al., 2015). LOC is equipment that automates and comprehends several laboratory processes into a chip-sized system that is not larger than a few square centimeters (Syedmoradi et al., 2021; Manessis et al., 2022). By altering reagents on the microscale, one may utilize phenomena such as fast heating and mixing. Additionally, it minimizes the waste and exposure to unpredictable chemicals. Several ultrasensitive tests have been established over the past 5 years, including nanotechnologies such as nanomaterials discovered in laboratories on-beads or surfaces using nanotechnology for laboratory use Enzyme-linked immunosorbent assay on a chip assays (ELISA) are at the core of their technology.

**FIGURE 1:**
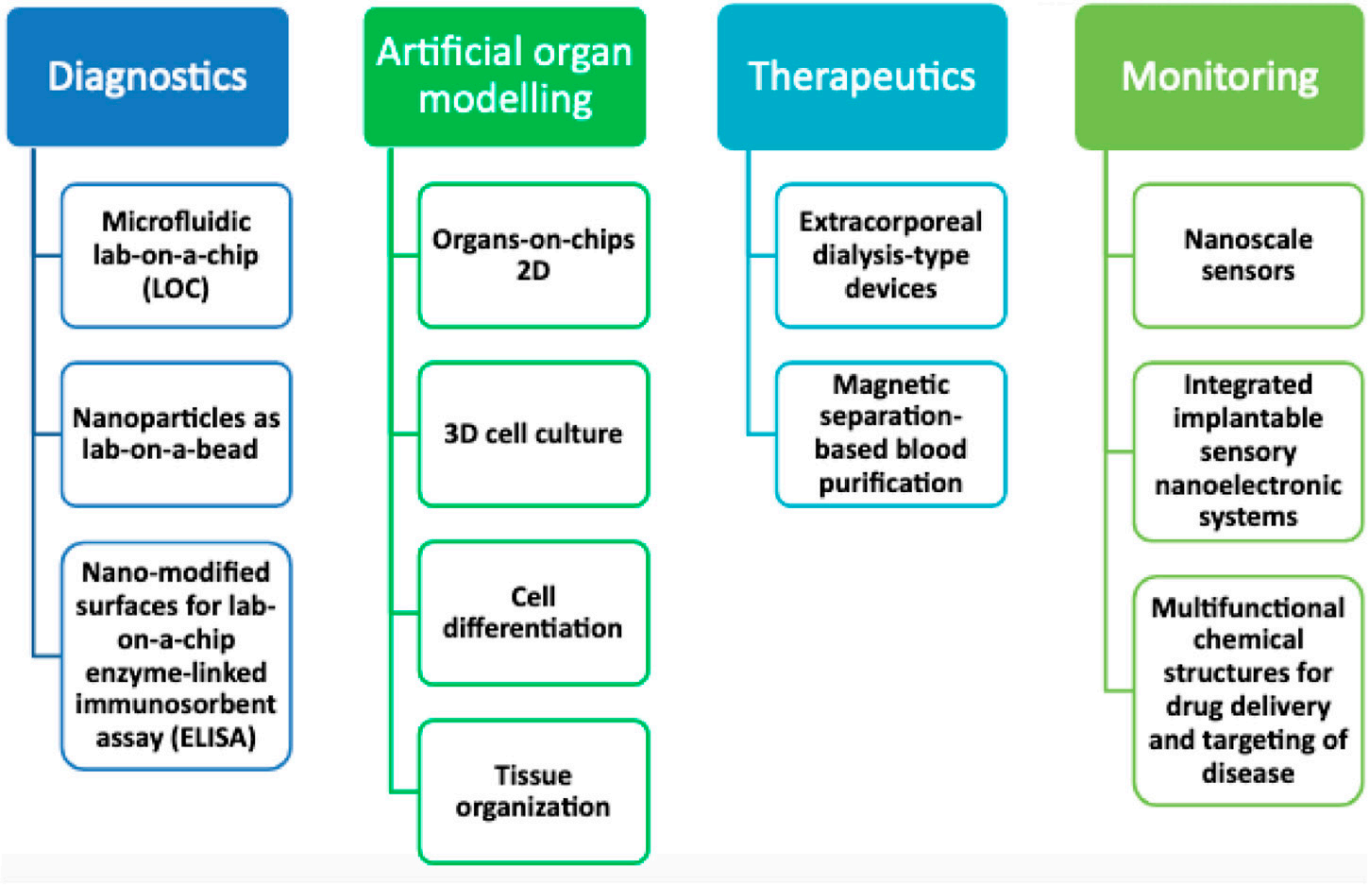
Future potential for nanotechnology advancement in critical care medicine study and experimentation in the next years.

Because there are not enough good clinical models, the pace of progress is slow in diagnostic and therapeutic research for several clinical disorders. This is especially applicable to those in critical condition. Currently, the most effective models are animal models. Animal models are inappropriate due to ethical problems and the inability to simulate human behavior effectively (Robinson et al., 2019). Reduced travel time between the laboratory and the patient’s bedside has been a focus in recent years, aiding in identifying disease causes and therapeutic targets. The convergence of LOCs and cell biology has made it possible to investigate the physiology of humans in an organ-specific set, hence establishing a unique model of multicellular human beings *in vitro*. The ability to investigate the illness locally, or even on the nanoscale, may improve our knowledge of the illness and yield intense frequent insights into certain patients that cannot be gained by employing animal models to reflect single-organisms clinical scenarios adequately. Organs-on-chips (OOCs) could permit accurate modeling of all biological processes of full living organs containing dynamic mechanical characteristics and biochemical functions (Puschhof et al., 2021). Particularly significant research based on unfulfilled needs would enable the development of improved clinical characterization and individualized therapy methods with new compounds for organ failure detection and local modulation. Incorporating OOCs into investigation employing by encouraging higher degrees of tissue structure and cell proliferation, 3d tissue models will implement and take over the 2D culture techniques (M. Singh et al., 2017). Acute respiratory distress syndrome (ARDS) could take advantage of this advancement because we will be capable of imitating the cellular features of an organ in a variety of ways, containing tissue-to-tissue interactions (such as vascular and epithelium-endothelium), spatiotemporal chemical gradients, and mechanically active microenvironments (for instance, arteries’ vasodilator and vasoconstriction reactions to fluid responsiveness, modifications in microcirculation (Beitler et al., 2016). The use of microfluidics in OOCs not only permits the evaluation of the effective transport and delivery of nutrients but also of other soluble signs across live 3D tissue constructions. In the coming years, Microfluidic high-throughput technology will be used to create medical devices-on-chips and OOCs, which will result in significant time and cost savings compared to test platforms currently being used. This will help with illnesses involving organ function, such as those affecting the heart, lungs, kidneys, and skin (Figure 1) (Sakr et al., 2012). To meet the demand for patient care, nanotechnology-based disinfectants need to be further advanced with optimum approaches. This review is aimed to discuss the increasing role of nanotechnology as an antimicrobial in various infectious diseases, including against the recent SARS-CoV-2. Moreover, we will also discuss the current challenges and future perspectives. This review will update the healthcare provider on the emerging use of nanotechnology-based antimicrobials. It will provide an overview of how it will shape the future of the healthcare system.

The introduction of fundamental nanotechnology principles in an accessible format, with examples of current clinical infectious diseases and public health applications, is critical, which will provide a foundation and aid understanding of and appreciation for this burgeoning and vital field of science. Herein, we review the current burden of infectious diseases, focusing on SARS-CoV-2 and bacterial infection, which are significant burdens on developed healthcare systems and small healthcare communities. We then highlight how nanotechnology could aid in improving existing treatment modalities and diagnosis of those infectious agents. In addition, we also highlight the kind of nanomaterials and their specific role in treating infectious diseases. Finally, we conclude the current development and future perspective of nanotechnology for combating infectious diseases. The overall goal is to update healthcare providers on the existing role and future of nanotechnology in tackling those common infectious diseases.

## 2 Nanotechnology-based bactericide and SARS-CoV-2 sensors in the healthcare system

**FIGURE 2:**
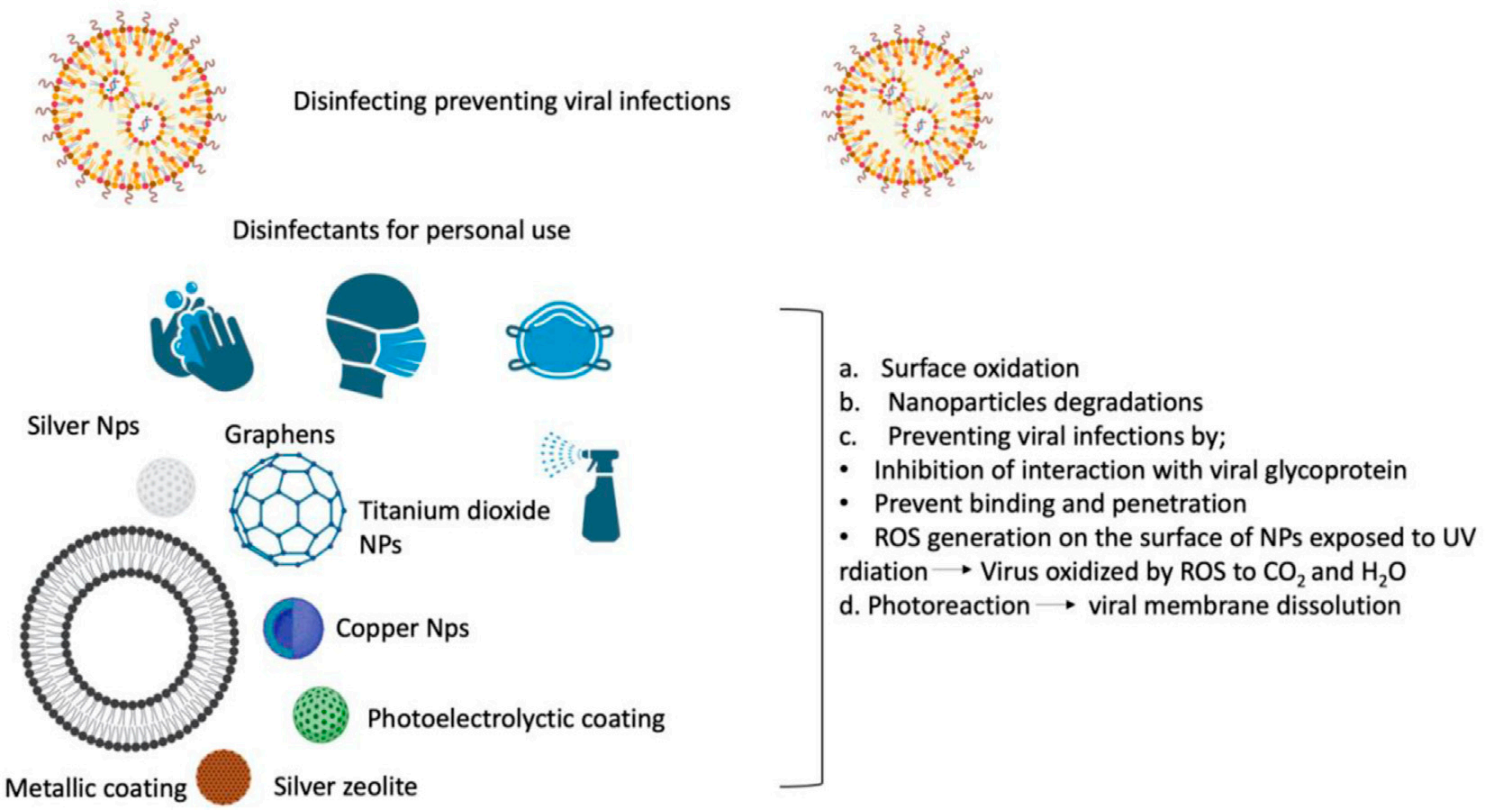
Viral disinfectants based on nanotechnology protect against SARS-CoV-2 by preventing viral propagation on surfaces, in the air, and in safety equipment. .

We have learned from the present coronavirus disease 2019 (COVID-19) epidemic how limited our ability to combat respiratory viral infections is. So far, SARS-CoV-2 has infected people in over 215 countries, infected over 674,003,861 individuals, and killed around 6,862,846. (19 2 February0,203, Johns Hopkins University Coronavirus Resource Center). Our immune system is our most crucial line of protection; On the other hand, immunocompromised individuals who have at least one underlying co-morbidity (such as heart disease, diabetes, or other underlying chronic health problems) are highly susceptible. Their primary defense line comprises hand sanitizers, immune system boosters, face masks, and clinically authorized medications (Hu et al., 2020). Antiviral materials and coatings based on nanotechnology may be implemented to stop the spread of SARS-CoV-2 through infectious material (Imani et al., 2020). Researchers from around the globe have made auspicious advancements in the development of COVID-19 prevention strategies. However, the advancement of treatments or vaccines still faces obstacles, for instance, regulatory difficulties, large-scale manufacturing, and public deployment (Dourado, 2020). The worldwide reaction will take months before this epidemic can be developed. In addition, we must be ready for the possible emergence of a second and maybe a third wave of the virus, demanding the development of alternative strategies to strengthen our immune system against not just COVID-19 but other viral infections with the potential to develop pandemics. The current state of our technological breakthrough, particularly in nanotechnology, is the solution to this problem. Significant research has been devoted to advancing Nanotechnology-based vaccines or antiviral drugs to fight SARS-CoV-2; however, none are available at the general level because of lengthy and stringent controlling processes. (Itani et al., 2020). Considering the different modes of coronavirus transmission (through cough or biofluids, or respiratory droplets), one method for combating the virus is to limit its spread by sanitizing the air, skin, and nearby surfaces (Figure 2).

Personal protective tools and surfaces have been disinfected and sterilized with chemical disinfectants (for example, peroxides, chlorines, alcohols, and quaternary amines), which are effective against various diseases (Fathizadeh et al., 2020). Most chemical disinfectants have limitations, including the necessity for high concentrations to eliminate the viruses, a gradual decline in effectiveness over time, and significant risks to the environment and human health. Correspondingly, metallic nanoparticles (NPs) (such as copper, silver, and titanium dioxide (TiO_2_). Metallic nanoparticles have fascinated scientists for more than a century and are now widely used in biological sciences and engineering. They are fascinating because of the tremendous potential they have for nanotechnology. Today, these materials can be synthesized and modified with a variety of chemical functional groups, allowing them to be conjugated with antibodies, ligands, and drugs of interest, thereby expanding their potential applications in biotechnology, magnetic separation, and preconcentration of target analytes, targeted drug delivery, and vehicles for gene and drug delivery, and most importantly, diagnostic imaging (Mody et al., 2010). NPs have been suggested as replacements because of their intrinsic wide range of antiviral and antimicrobial efficiency, longevity, and effectiveness at far lower dosages (Shahzadi et al., 2018; Sportelli et al., 2020). For example, preliminary tests revealed that a silica composite/silver nanocluster covering on face masks has antiviral properties against SARS-CoV-2 (Balagna et al., 2020). NanoTechSurface of Italy created a self-sterilizing solution for surface disinfection made of TiO_2_ and silver ions. Similarly, FN Nano Inc., United States, created a photocatalytic coating (light-mediated) built on TiO_2_ nanomaterials that, when subjected to light, can damage organic compounds that have viral barriers on their surface by degrading the chemical compounds. To strengthen their inhibitory effect, NPs could also be included in respiratory face masks (Weiss et al., 2020). A study performed at the Queensland University of Technology in Australia has created a biodegradable cellulose nanofiber filter cartridge capable of filtering particles smaller than 100 nm (Widdowson, 2020). Additionally, because of their distinctive chemistry, physical, and surface-to-volume ratios, graphene NPs could be used to adsorb and remove SARS-CoV-2 (Palmieri and Papi, 2020). LIGC Applications Ltd., US, has created a recyclable mask comprising microporous, electrically-conducting graphene foam that attracts and kills bacteria (Talebian et al., 2020). Nanomaterials based on graphene are potential biomedical applications such as personal protective equipment against the ongoing COVID-19 outbreak (Er et al., 2022). One of the previous studies reported that ferroptosis contributed to RSL3-induced cell death in CRC cells, and ferroptosis may be a widespread and dynamic form of cancer cell death and other diseases (Sui et al., 2018).

## 3 Nanotechnology in wound healing

In tissue regeneration, an increasing number of cutting-edge nano therapies are now being studied in a clinical trials. Several nanoscale techniques, comprising nanoparticles, were investigated for targeting distinct stages of wound healing (Figure 2). There are 02 types of nanomaterials exploited in the healing process: 1) nanomaterials with inherent characteristics useful in the treatment of wounds and 2) NPs are used as delivery vehicles for pharmaceutical agents. (Tocco et al., 2012; Palmieri and Papi, 2020).

### 3.1 Metal oxide and metallic nanomaterials

Silver is known for its antimicrobial properties, arbitrated *via* inhibiting respiratory enzyme pathways and the modification of microbial cell walls and DNA. The effectiveness of Silver against biofilm-forming and multi-resistant bacteria is beneficial for its usage for wound healing. Bacterial biofilms and enclosed bacterial colonies in extracellular polymeric self-production material for protection characterize chronic injuries. It has been demonstrated that wound-associated biofilms trigger apoptosis and the release of ROS. This results in the generation of inflammatory cytokines that contributes to persistent inflammation and inhibit re-epithelialization. Furthermore, biofilm bacteria provide a therapeutic challenge because of their perseverance and resistance to standard antibiotic treatment. Compounds of silver, for example, silver sulfadiazine and silver nitrate, are widely used to cure chronic wounds and burn infections. Non-etheless, some chemicals, mainly silver sulfadiazine, may cause tissue damage. To avoid this disadvantage, silver nanoparticles (Ag-NP) with a surface-to-volume ratio was high, making these substances more proficient at low concentrations and reducing their toxicity compared to conventional silver compounds. Ag NPs can cause harm to the internal and extracellular components of bacteria, which enables them to have a broad-spectrum antibacterial action (Zheng et al., 2018). Pure silver nanoparticles have been shown to cure inflammation *via* cytokine regulation and promote wound healing with reduced scar formation. Ag-NP comes in various shapes and sizes with varying degrees of antibacterial and antimicrobial activities that were also researched and shown. Kelestemur et al. reported that thiolated oligonucleotide surface alteration of Ag-NP (5′-HS-(CH2)6TAATGCTGAAGG-3) decreased the cytotoxic impact by extending the release of silver ions. Because of its effectiveness in releasing clusters of Ag-NP that may cover a large wounded surface area, the nano-crystalline silver form of silver is also regarded as beneficial (Dunn and Edwards-Jones, 2004). Dressings made of nanocrystalline silver such as Acticoat enabled prolonged release of Ag+, as a result, interacting with target tissues or protein complexes in wound fluid to prevent possible silver consumption. Non-etheless, there are still disadvantages to employing silver-based nanoparticles for tissue regeneration, such as skin staining to a blue-gray hue and the reappearance of germs resistant to silver. (Nascimento et al., 2009; Peterson, 2009; Rice, 2009). Against microorganisms, it is reported that AgNPs/TA in combination with antibiotics may have a therapeutic application. In addition, these compressed nanoparticles have excellent antioxidant activity and can be used in various biomedical science disciplines (Begum et al., 2022). The effect of metals on the activity of NPs is crucial before subjecting them to practical application. For instance, when prepared under the same condition, the AuNPs degraded the dyes in a shorter time as compared to the AgNPs, though both are stabilized (Megarajan et al., 2022).

### 3.2 Non-metallic nanomaterials

As restorative agents for wounds, non-metallic nanomaterials were proposed. Fullerenes, carbon nanotubes, and graphene are carbon-based nanomaterials that have shown tremendous promise to be used as nanomedicine in a variety of biological applications, including bioimaging, tissue regeneration, and controlled pharmaceuticals (Zhou et al., 2010). Fullerenes exhibited promising results for wound-healing applications (Zhou et al., 2010) due to their antioxidant and anti-inflammatory properties. Fullerenes are excellent for applications that require wound healing because of their antioxidant abilities to scavenge and detoxify reactive oxygen species (ROS) and reactive nitrogen species. If fullerenes are linked to cells, they may group in the biological system.

Nanomaterials based on carbon have been presented as potential wound-healing agents. Carbon fullerenes possess intriguing features that prevent numerous pathogenic pathways responsible for impeding wound healing. Some fullerene compounds have shown anti-inflammatory and antioxidant capabilities, designating them as wound-healing medicinal agents. Recent research has demonstrated the photothermal therapy of microbial and fungi-infected wounds using graphene oxide (GO) nano-sheets. The heat produced when these nanoparticles were subjected to a near-infrared Nd:YAG laser-assisted GO’s anti-infection properties. Infected wounds healed more rapidly when treated with laser and GO, giving a non-invasive alternative to antibiotics.

Polymeric materials have wound-healing and antibacterial properties, NPs, mainly found in natural polymers such as chitosan NPs, have been investigated. Polymeric nanomaterial treatment employs polymeric ingredients as dressings or delivery vehicles. Due to its biodegradability, biocompatibility, hemostatic action, and antimicrobial qualities, as a dressing material, the natural polymer chitosan was used (Archana et al., 2013). Chitosan composites typically exhibit features that neither chitosan nor the added elements exhibit on their own (Patrulea et al., 2015).

Black phosphorus (BP) has recently emerged as an outstanding nanomaterial for numerous biomedical applications, including wound healing. Huang et al. (2018) According to a recent study, water-derived BP nanosheets could be employed as a moldable substrate for applications including wound healing. These BP-based materials demonstrated outstanding biocompatibility with NIR-mediated photothermal effects that effectively prevented bacterial infections and subsequently promoted wound healing; this indicates immense clinical potential for the treatment of wounds. Moreover, Mao et al. (2018) reported that BP-embedded hydrogel has a high potential for wound healing and sterilization. The capacity of BP-based nanomaterials to produce singlet oxygen (1O2) under simulated visible light, led to faster bacterial death. During the wound-healing process in rodents, this hydrogel has high reproducibility in terms of antibacterial activity and biocompatibility without causing significant defects or harm to major organs such as the heart, liver, pancreas, lung, or kidney. Therefore, the nanocomposite made of BP may be used in photothermally mediated antibacterial activity and wound healing (Mao et al., 2018).

Among polymeric nanoparticles, Poly(lactic-co-glycolic acid) (PLGA) has been extensively investigated for the wound-healing process (Chereddy et al., 2016). It is a suitable nanomaterial for applications involving wound healing due to its biodegradability and biocompatibility, which are similar to those of liposomes. Research has examined the antibacterial activity of antibiotics encapsulated in PLGA against *Escherichia coli*, *Staphylococcus aureus*, and *Pseudomonas aeruginosa*. As an alternative to polymer and liposome nanomaterials, a new class of nano-carrier and lipid-polymer hybrid nanoparticles recently emerged (Cheow and Hadinoto, 2011; Hadinoto et al., 2013). These nanoparticles incorporate the benefits of liposomes and polymeric nanoparticles. In addition, nanoparticle-functionalized antibiotics may facilitate wound-healing applications. Chen et al. (2015) demonstrated that functionalized gold nanodots containing antimicrobial peptides inhibit the growth of drug-resistant bacteria and promote wound healing in a rodent wound model. In a separate study, the combination of Au NPs with epigallocatechin gallic acid and -lipoic acid accelerated wound repair in diabetic ulcers (Chen S. A. et al., 2012). In this regard, the formulation of solid-lipid nanoparticles with Q10 for topical therapeutic or cosmetic objectives is available on the market as the NanoRepair Q10^®^ product by Dr. Rimpler (Souto and Müller, 2005).

## 4 Nanoparticles as antimicrobials

The development of nanotechnology in the 1980s made Nano-scale manufacturing possible by monitoring atomic particles. Consequently, nanotechnology has gained significance in various fields, including organic chemistry, biomaterials, and medication. Nano-scale and nano-medicine particles (Medina et al., 2007) are utilized for therapeutic reasons, such as biomaterials, and as diagnostic instruments (Surendiran et al., 2009) in the fields of medical research and healthcare. Thus, medicinal applications for future molecular nanotechnology and NPs are abundant (Freitas Jr, 2005; Wagner et al., 2006). Nanotechnology has made numerous challenging diagnoses feasible and increased our understanding of the cause of illness. Due to their diminished efficacy, utility, and bad effects, several drugs and treatments are critically compromised. Since NPs are about the same size as biomolecules and components, *in vitro* and *in vivo* treatment techniques with greater specificities (Medina et al., 2007) are advantageous. Medications with a higher specific activity can boost efficacy and minimize unwanted effects. In high-risk locations, nanoparticles with a smaller size may be used for therapy, lowering the possibility for injury and providing the exact quantity of drug necessary.

Despite its previous therapeutic efficacy, antibiotic use became problematic from the start of the 20th century (Cohen, 2000), due to the advent of germs resistant to drugs (Ding et al., 2021). Augmented pathogen resistance to colistin (Pitt et al., 2003), tigecycline (Sheng et al., 2014), and carbapenems (N Ramesh et al., 2004; Ramesh et al., 2008), as well as stopped creation of novel antibiotics, rendered it hard to treat the infectious illness caused by hazardous microorganisms. Even if a novel antibiotic is discovered, there is no assurance that it will be effective against all multidrug-resistant illnesses (Rice, 2009). Yet, gram-positive and gram-negative microorganisms are evolving rapidly than before, posing serious risks to the public’s health. (Boucher et al., 2009). Therefore, technical developments must support the long-term need for adequate treatment of bacterial infectious diseases that are resistant to drugs and aid in the fight against these harmful infections. (Taylor et al., 2002).

The most recent advancements in creating medical NPs might favor antibiotics’ function. NPs, according to the hypothesis, are a contemporary class of synthetic pathogens and antimicrobials. It is possible that using NPs as antibiotics will lower resistance and enhance antibiotic delivery (Rai et al., 2009). The latest research indicates that some antimicrobial-activated metal nanostructures have indeed assisted in the fight against infectious illnesses (Goodman et al., 2004). These structures have lesser toxicity, lower cost, and improved pharmacokinetic parameters, and they aid in eradicating drug-resistant bacteria. The key benefit is that they are more potent than traditional antibiotics for a longer period of time, which is excellent for a long-lasting, sustained therapeutic impact (Pal et al., 2007).

Antimicrobial resistance has presented a worldwide threat to humanity that is exceptional. Traditional antibiotics are reducing their efficacy against many drug-resistant bacteria and are constantly developing. Future nano-antibiotics (nAbts) are being developed using a pharmaceutical delivery method based on nanotechnology, and it is regarded as the technology revolution’s 21st-century weapon. The recently created discipline of nanotechnology remains in its emergence, requiring colossal effort and expense for the evolution of newer pharmaceutical medications throughout time. Nanoparticles have various advantages over conventional antibiotics, including absorption, durability, controlled release, dispersion, and administration. Using pressurized gyration, antibacterial polymer nanocomposite fiber meshes incorporating graphene oxide (GO) nano-sheets were successfully produced. The ability of GO nano-sheets placed in a polymer matrix to preserve their antibacterial characteristics makes them a promising antibacterial agent (Matharu et al., 2020).

Moreover, NPs can be economical, environmentally friendly, and adaptable to the resilient, antibacterial environment. Nanoparticles possess different and well-defined chemical and physical properties that may be adjusted for specific applications. In addition, their superior antibacterial activity results from their large volumetric surface area, giving them an edge over chemical equivalents experiencing drug resistance issues. NPs and nanotechnology synthesis advancements must open the floodgates to develop innovative ways to create novel antibacterial agents. Numerous techniques with varied characteristics, such as morphology, size, surface coatings, and electrical charge, enable investigators to generate unique composite antimicrobial compounds for various antimicrobial applications. The antibacterial activity of inorganic NPs and carbon-based NPs will be used in future medical, scientific, and industrial research and development to address the problem of antimicrobial resistance to standard treatment methods. Nanoparticles also offer a variety of options for managing biofilms as well as for preventing and detecting infections. Before these NPs were widely used in business, however, a detailed investigation was necessary to determine their impacts on naturally occurring organic tissues and how they affect people and the environment.

## 5 The role of nanotechnology in nosocomial infections

The development of new techniques and therapeutic innovations have received constant attention over the past decade. In the post-antibiotic era, managing multidrug-resistant (MDR) pathogens is vital. A promising solution has been identified in nanotechnology. According to the BCC analysis, the global market for healthcare-acquired infections (HCAIs) will increase from $18.9 billion in 2018 to $24.7 billion in 2023. The nanomedicine market will increase from $151.9 billion in 2017 to $293.1 billion in 2022 (Ananda et al., 2022). From Professor Richard Feynman’s conceptualization of nanotechnology in the 1960s through Drexler’s foresight of its usage in molecular biomedicine (Drexler, 1981), the effect of nanotechnology has raised tremendously in numerous fields. This review of the scientific literature reveals that nanotechnology will assist several medical sectors in the near future and extend fast into many other fields.

Antibiotic resistance is a growing concern in the modern world. In addition, genetic resistance to antibiotics is frequent in bacteria such as MRSA. Nanomaterials inhibit infection-causing bacterial growth. Nanoparticles penetrate bacteria and biofilm, causing the production of ROS that eradicates bacteria. As a result, NPs represent a revolutionary method for combating bacterial illnesses resistant to antibiotics. Several nanomaterials, like NPs and nanotubes, are used immediately in biomedical equipment to prevent the transmission of disease.

**FIGURE 3:**
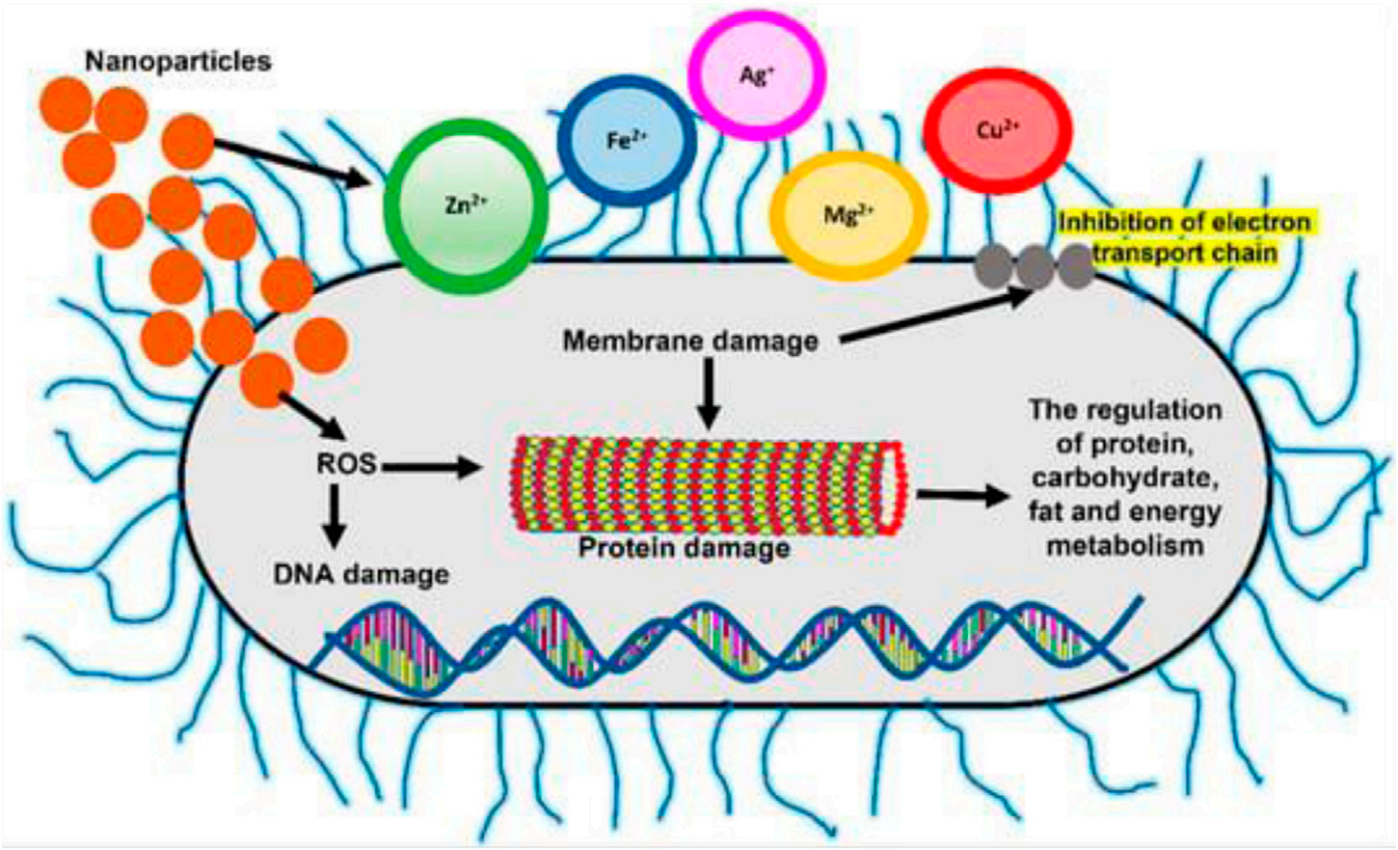
Changes in free radical generation and membrane permeability, which cause protein and DNA damage, are two mechanisms of NPsactivity in bacterial cells (L. Wang et al., 2017).

Due to their small size and high surface area to volume ratio, NPs interact with bacterial cells far more favorably than microparticles do. As depicted in Figure 2, these NPs provide various interactions between cell walls and nanomaterials, for instance changing the permeability of membrane by permeation, inhibiting oxidative phosphorylation, or producing free radicals that harm the cell membrane and triggering cell death, consequently raising oxidative stress and damaging DNA (R. Singh et al., 2014). In addition, the ionic activity of NPs can affect the bacterial signal transmission, inhibiting bacterial growth or inactivating enzymes through interaction. There are other physical contact mechanisms, such as bacteria covering, to create surface tensions as well as permeation *via* sharp edges, which cause physical harm and negative chemical consequences. The creation of anti-adhesion surfaces that stop the growth of biofilm is also beneficial (Bregnocchi et al., 2017). In this research, an effort was made to emphasize the possible unique solutions provided by nanotechnology for nosocomial infection prevention, MDR strain control, and disease diagnosis (Figure 3).

The rising occurrence of antimicrobial resistance has urged us to seek antibiotic alternatives. Nanotechnology aided in the development of NPs with several functions that provide a variety of viable answers to the global nosocomial infection concern. Its expanded usage can aid in the reduction of nosocomial infections in the healthcare industry. Currently, bottleneck concerns such as possible toxicity and the effect of long-term contact with NPs with decreasing diameters on the normal cell function prevent the widespread application of nanotechnology solutions. MDR strains are resistant to these innovative technological solutions and their effects, yet all these problems are resolvable. Despite several indications of development, solutions based on nanotechnology are not commonly acknowledged in treatment, diagnosis, and control due to their high cost, lack of information regarding product functionality for medical professionals, and worries patients. Moreover, when the same NPs that are effective in curing MDR strains *in vitro* are utilized, there is a strong likelihood that outcomes may vary. They all reflect shortcomings in understanding the needs, consequences, and outcomes of *in vivo* and *in vitro* surroundings.

An earlier study compared the administration of benzalkonium chloride (BC), a nanotechnology-based product, for 24-h periods *versus* didecyl dimethyl ammonium chloride (DDAC) for 12-h periods in terms of the efficacy of surface antiseptics in the ICU. BC, a product derived from nanotechnology, leaves its active metabolites on the surface; it is applied by forming a sponge-like layer. Since the effectiveness of BC lasts for 24 h and is used to perform cleansing at 24-h intervals, we believe it is more cost-effective and beneficial in terms of workforce gain.

## 6 Bio-Kil nanotechnology-based antimicrobial approach used in ICU

Hospitals confront rising expenditures when more people develop healthcare-associated diseases (HAI) and hospital stays increase (Haley et al., 1985a; Haley et al., 1987). The transmission of HAI infections occurs by air, droplets, and staff hand contact. It has been determined that equipment contamination is a significant source of the bacteria implicated in HAI (Boyce, 2007; Oie et al., 2007). According to the research (Hardy et al., 2006; Oie et al., 2007), rooms’ surfaces of hospitalized individuals infected or colonized with vancomycin-resistant enterococci (VRE), MRSA or *S. aureus* is more likely to become contaminated by these germs. This shows that patients can transfer diseases such as MRSA and VRE to their surroundings. Even though hand hygiene has been a priority in hospitals across the world, further measures are needed to reduce the colonization density in the hospital context, which is projected to have a major influence on reducing HAI incidence to break the chain of infection (P.-R. Hsueh et al., 2014; Pritchard and Raper, 1996).

Bio-Kil (Cargico Group, Taiwan) is a quaternary ammonium compound (QACs) antibacterial agent composed of inorganic metal materials. Pathogens are drawn to Bio-Kil molecules due to their high binding structure and strong electric field. Their powerful electrical charge destroys the microbes’ membrane proteins, killing germs (Hsueh et al., 2014). Bio-Kil develops a covalent, permanent connection with the textile fiber surface. Even after 50 launderings, the product retains more than 90 percent of its antibacterial effectiveness (Chen Y. L. et al., 2012). Based on how often textiles are washed, the treatment should be repeated every three to 6 months to maintain long-lasting bactericidal activity (Chen Y. L. et al., 2012). Similarly, The Bio-Kil^®^ anti-bacterial catalyst creates a direct covalent bond with the surfaces of the ICU’s equipment, including workstations, nurses’ stations, desktops, computer keyboards, phones, and surfaces close to patients, where it provides a long-lasting bacterial activity. Lower bacterial counts in the hospital’s environment lessen the chance that equipment, surroundings, and linens will become contaminated, which lessens the risk of HAIs brought on by bacterial colonization (Chen Y. L. et al., 2012).

**FIGURE 4:**
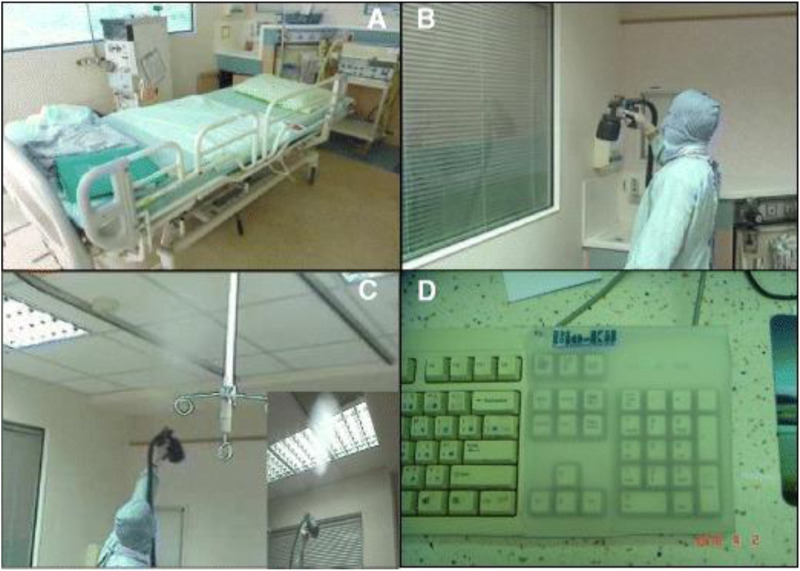
Application of Bio-Kil bacteria killing nanotechnology in the intensive care unit. **(A)** Ten sets of new textiles (pillow cases, bed sheets, duvet covers, and patient clothing) were provided by the researchers for both S-19 and S-20. Clothing for family members, nurses, and doctors were routinely provided by the hospital. All the textiles placed in S-20 were treated by Bio-Kil solution. **(B)** All room walls in S-20 were treated evenly with Bio-Kil solution. **(C)** Bio-Kil solution was sprayed evenly on the air filter and the ceiling in S-20. **(D)** A Bio-Kil antibacterial silicon pad (50 cm × 50 cm) was placed over the instrument panel and computer keyboard in the nursing station in the S-20 ward. Reproduced with permission from (Hsueh et al., 2014).

**FIGURE 5:**
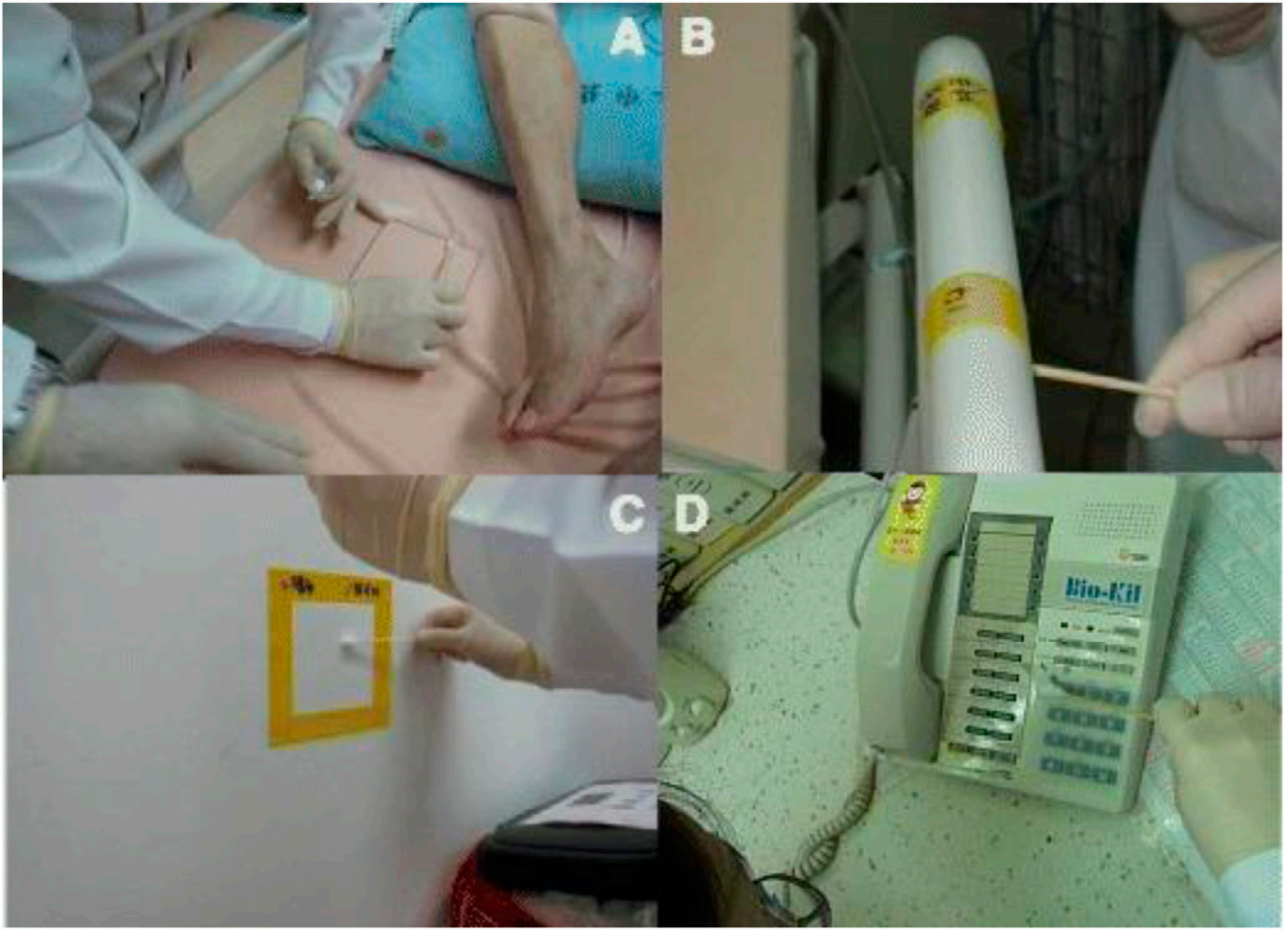
Bacterial culture sampling by swabbing a 10 cm × 10 cm square of **(A)** bed sheet, **(B)** bedrail, **(C)** a telephone keypad and cement wall enclosed with a Bio-Kil silicon pad. **(D)** A telephone keypad covered with a Bio-Kil silicon pad. Reproduced with permission from (Hsueh et al., 2014).

An internal air conditioning system circulates air in the ICU’s air filtering system 8–12 times per hour. When air moves through a system treated with Bio-Kil, it contacts the platform, where a catalytic reaction will eliminate the bacteria (P.-R. Hsueh et al., 2014). So, as it circulates, the air is continuously cleaned, decreasing the number of microorganisms (Figures 4, 5).

Bio-Kil substantially decreased the number of microorganisms in the ICU. Maintaining a secure environment and disease control strategies in healthcare settings is crucial to avoid HAI (Yang et al., 2001). The bulk of HAIs is produced by colonization as well as diseases induced by endogenous flora, whereas foreign environmental entities have been neglected as an unimportant risk for HAI for decades (Weber et al., 2010; Passaretti et al., 2013). Meanwhile, HAI brought on by exogenous infections is significantly influenced by cross-transmission resulting from direct physician-patient interaction. (Weber et al., 2010), Washing hands has been the most critical and preventive measure in HAI care since Semmelweis’ time (Pritchard and Raper, 1996). In actual practice, however, a compliance rate of 30% has always been a challenge (Pritchard and Raper, 1996; Yang et al., 2001).

The hand washing promotion was shown to be the most cost-effective and most acceptable of the several contagion control packages and implemented zero-tolerance rule following SARS (Haley et al., 1985b; Yen et al., 2011); it has also contributed to a tendency toward a decline in nosocomial MRSA infections (Tseng et al., 2012). There has been an increase in the frequency, in recent years, of MDR epidemics, for instance as carbapenem or pan drug resistance Vancomycin-resistant enterococci or *Acinetobacter* baumannii (Hardy et al., 2006; P.-R. Hsueh et al., 2014; Hsueh et al., 2002; Oie et al., 2007; Weber et al., 2010). Research has shown that HAI Unintended interaction with the patient’s or the hospital’s circumstances led to transfer from doctors to patients’ environments. Ohl et al. (2012) hypothesized a relationship between the transmission of microorganisms to patients and medical staff (HCWs) who do not wash their hands after handling fabrics. Recently, environmental disinfection has been stressed to compensate for poor hand hygiene practices. Along with lessening the possibility that MDRO will be transmitted from a previous occupant to a new tenant, the area is cleansed when each patient leaves. (Nseir et al., 2011; Passaretti et al., 2013), Environmental cleansing of a patient’s bed and surroundings is crucial to managing MDR (P.-R. Hsueh et al., 2014; Weber et al., 2010). Though, there has always been a danger of quality instability in manual surface washing and disinfection due to inadequately managed procedures or, potentially, the probably undertrained hands of the house working bodies (P.-R. Hsueh et al., 2014; Weber et al., 2010).

The number of germs in the environment and the prevalence of infectious diseases (including air, surfaces, and fabrics) can be significantly reduced by using Bio-Kil nanotechnology or colonization among ICU patients.

## 7 Concluding remarks and future perspective

Detection, treatment, and prevention of infectious diseases have been vexing challenges to humankind since our origin. Nanotechnology brings significant benefits for diagnosing and treating cancers. With the success of NPs in gene therapy and cellular therapy against cancer, researchers are actively working on expanding the therapeutic avenue of NPs beyond cancers. In an effort, one potential arena is the role of nanotech as an antiseptic for various infectious diseases in the healthcare system. For instance, a silica composite/silver nanocluster covering on face masks has antiviral properties against SARS-CoV-2. Various kinds of metallic and non-metallic NPs are increasingly used for wound healing and treating wound-related infections. Bio-Kil is an antimicrobial consisting of inorganic metal components and organic components, was reported to decrease the bacterial environmental burden and multidrug-resistance organisms in ICUs.

Nanotechnology brings significant benefits for diagnosing and treating cancers.

So far, various dialysis strategies are adopted to remove toxic substances from the body in the ICU and healthcare system. The key focus of this approach is high-grade safety with minimum toxicity (Wu et al., 2011). To collect and accurately measure minimal quantities of cellular, protein, or nucleic acid biomarkers, highly selective functionalized surfaces created by nanotechnologies will be at the center of this transition (V. Wong et al., 2011). Nanoscale detectors provide remarkable temporal and spatial resolutions, or embedded microfluidic gadgets do “liquid biopsies” swiftly and supply the clinician with the data necessary to alter treatment. Combining these chemical findings with more prevalent physical measurements like respiration rate, temperature, cell oxygenation, breathing, and microcirculation provides exceptional insight into a patient’s condition at the cellular, molecular, and tissue levels. These quantitative research data links are made *via* closed-loop feedback control systems, anesthetic, surgical, and “electro pharmaceutical” interference.

To precisely target the area of activities and deliver their therapeutic payload there at the necessary rate, “Nanobots” circulate in the blood like “Exocet” rockets, minimizing cell collateral damage caused by the harmful consequences of the pharmaceuticals (P.-R. Hsueh et al., 2014). This supply is initially based on the region and amount of a suitable biomarker. This capability is provided in a novel way by nanostructured, “biofunctionalized” interfaces made of “smart” 4D materials (structures that can be changed in a pre-programmed manner in response to a stimulus; Hsueh et al., 2002). These interfaces are fully biocompatible and have properties that can evolve over the period in response to modifications to the surroundings surrounding them. One example is the creation of surfaces with nanoparticles that regulate the blood’s bio-compatibility in extracorporeal circulation (J. L. Wang et al., 2009). Future advancements are anticipated in medicine delivery, cell healing, artificial organ creation, etc. Most of the currently available marketed uses of nanotechnology in medicine are aimed at the delivery of drugs, allowing new mechanisms of action along with enhanced targeted and bioavailability of known medicinal elements, and implementation risks abound. In the near future, novel nanotechnological applications, such as nanostructures that enable transportation through biological boundaries, fully implanted sensory nanoelectronic systems, remote control of nanoprobes, and multipurpose chemical structures for medication delivery and ailment targeting are all required.

The level of interest and pace of nanomaterials research, as well as the biophysical alteration of exposed surfaces of living tissue to restrict the spread of infection, is a source of optimism. To better understand the interactions among coated biofilms, hosts, and surfaces, it is now a requirement of time to gather more knowledge on the toxicology and pharmacokinetics of NPs *in vivo* applications. Further research is required before any clinical use. Nanoengineers and microbiologists must collaborate to develop more effective and widely accepted therapeutic options against MDR strains. Once achieved, it will pave the way for effective deployment on a greater scale in the clinical sector. In addition, cost-effective solutions must be discovered for the betterment of humanity. This literature review aims to raise awareness of the scope of the problem posed by nosocomial diseases and the possible role nanotechnology might play in combating them.

Recent papers have highlighted the development of numerous nanotechnologies, remarkably multifunctional technologies, in nanotherapeutic treatments and wound healing. It is difficult to understand the physicochemical characteristics of nanoscale systems, their predicted behavior, and their toxic effects on the human body. Furthermore, the FDA’s demand for high purity in scaffolds and NPs approved for human use presents a challenge because bulk synthesis and NP and polymer purification are usually challenging. Therefore, there is an ongoing requirement for improved analytical techniques and artificial devices to translate nanotechnology-based techniques into clinical practice. Many attempts are also required to equip persistent wound therapies with respect to location and targeted efficacy to prevent undesired occurrences and interventions that could impede the biological functioning of nanosystems in the human body.
